# Prognostic value of the extent of lymphadenectomy for esophageal cancer-specific survival among T1 patients

**DOI:** 10.1186/s12885-021-08080-4

**Published:** 2021-04-14

**Authors:** Yang Wang, Xiangwei Zhang, Xiufeng Zhang, Jing Liu-Helmersson, Lin Zhang, Wen Xiao, Yuanzhu Jiang, Keke Liu, Shaowei Sang

**Affiliations:** 1 Department of Medical Imaging, Shandong Provincial Hospital Affiliated to Shandong First Medical University, Jinan, 250021 People’s Republic of China; 2 Department of Thoracic Surgery, Shandong Provincial Hospital Affiliated to Shandong First Medical University, Jinan, 250021 People’s Republic of China; 3 Department of Respiratory and Critical care, Shandong Public Health Clinical Center, Jinan, 250013 People’s Republic of China; 4 Centre for Sami Research, Umea University, SE-901 85 Umea, Sweden; 5 Shandong Institute of Clinical Medicine, Shandong Provincial Hospital Affiliated to Shandong First Medical University, Jinan, 250021 People’s Republic of China; 6 Clinical Epidemiology Unit, Qilu Hospital of Shandong University, 107 Wenhua Road, Lixia District Jinan, 250012 People’s Republic of China; 7 Clinical Research Center of Shandong University, Jinan, 250012 People’s Republic of China; 8 Department of Epidemiology and Health Statistics, School of Public Health, Shandong University, Jinan, Shandong People’s Republic of China

**Keywords:** Esophageal cancer, T1, Cancer-specific survival, Prognosis, Extent of lymphadenectomy, Cutoff value

## Abstract

**Background:**

Clinically, there are no clear guidelines on the extent of lymphadenectomy in patients with T1 esophageal cancer. Studying the minimum number of lymph nodes for resection may increase cancer-specific survival.

**Methods:**

Patients who underwent esophagectomy and lymphadenectomy at T1 stage were selected from the Surveillance, Epidemiology and End Results Program (United States, 1998–2014). Maximally selected rank and Cox proportional hazard models were used to examine three variables: the number of lymph nodes examined, the number of negative lymph nodes and the lymph node ratio.

**Results:**

Approximately 18% had lymph node metastases, where the median values were 10, 10 and 0 for the number of lymph nodes examined, the number of negative lymph nodes and the lymph node ratio, respectively. All three examined variables were statistically associated with cancer-specific survival probability. Dividing patients into two groups shows a clear difference in cancer-specific survival compared to four or five groups for all three variables: there was a 29% decrease in the risk of death with the number of lymph nodes examined ≥14 vs < 14 (hazard ratio 0.71, 95% confidence interval: 0.57–0.89), a 35% decrease in the risk of death with the number of negative lymph nodes ≥13 vs < 13 (hazard ratio 0.65, 95% confidence interval: 0.52–0.81), and an increase of 1.21 times in the risk of death (hazard ratio 2.21, 95% confidence interval: 1.76–2.77) for the lymph node ratio > 0.05 vs ≤ 0.05.

**Conclusions:**

The extent of lymph node dissection is associated with cancer-specific survival, and the minimum number of lymph nodes that need to be removed is 14. The number of negative lymph nodes and the lymph node ratio also have prognostic value after lymphadenectomy among T1 stage patients.

## Background

The American Joint Committee on Cancer (AJCC) TNM Classification suggests that lymph node status is one of the most significant prognostic factors in esophageal cancer. For staging purposes, the National Comprehensive Cancer Network (NCCN) guidelines recommend that a minimum of 15 lymph nodes should be removed and examined [1]; however, for therapeutic purposes, the extent of lymphadenectomy remains under debate, especially in patients with T1 esophageal cancer.

Lymph node metastasis is a common mechanism of cancer progression in esophageal cancer. Lymph node metastases are thought to be very rare in patients with early esophageal cancer, but studies have shown that the prevalence of lymph node metastases in T1 patients ranges from 16.6% to nearly 40% depending on different histopathological characteristics [2–6]. Lymph node metastasis is known to influence the prognosis of esophageal cancer. Esophagectomy is the standard treatment for early stage patients, but surgeons still debate the prognosis of the extent of lymph node dissection [7–10], and the extent of lymph node dissection is still unclear in T1 patients.

The purpose of the study is to (1) investigate the prevalence of lymph node metastases; (2) explore the relationship between the extent of lymph node resection and long-term cancer-specific survival (CSS) in T1 patients after esophagectomy and lymphadenectomy; and (3) find the optimal or the minimum number of lymph nodes that need to be removed.

## Methods

Our study is based on the U.S. data from the Surveillance, Epidemiology and End Results (SEER) Program database (https://seer.cancer.gov/data/). The SEER database collects cancer incidence data from population-based cancer registries, which includes patient demographics, primary tumor site, tumor morphology, stage at diagnosis, and follow-up with patients for vital status. The ASCII text version of data from 1973 to 2014 (released in November 2016) was downloaded for this study. The SEER data are deidentified and publicly available. Therefore, the study is exempted from institutional ethical review board approval.

We identified all cases of first primary esophageal cancer (International Classification of Diseases for Oncology with tumor site codes 150–155, 158–159). Then, we selected patients diagnosed with esophageal adenocarcinoma (EAC) (SEER codes 8140–8389) or squamous cell carcinoma (ESCC) (SEER codes 8050–8089) between 1988 and 2014. Next, we narrowed the cases down to include only those patients who were treated with esophagectomy and lymph node dissection and survived more than 3 months after the operation. Other inclusion criteria were microscopic diagnostic confirmation, T1 stage and active follow-up (obtaining the outcome of patients after surgery at periodic intervals by contacting the patient, a physician, family member, or other informant). Tumors with distant metastasis were excluded.

Demographic, diagnostic and survival information was extracted, including sex, age at diagnosis, year of diagnosis, primary site, grade, histology, lymph nodes examined, positive lymph nodes, tumor size, cancer-specific death and survival time. The primary outcome of the cohort was the time from diagnosis to esophageal cancer-specific death. Cases with missing data were excluded from the study.

In the study, the number of lymph nodes examined (nLNE), the number of negative lymph nodes (nLNN) and the lymph node ratio (LNR = the number of positive lymph nodes/nLNE)] was used to define the extent of lymph node dissection. The statistical method, maximally selected rank statistics [11], was used to identify the optimal cutoff point for the three main variables: nLNE, nLNN and LNR. Then, we divided patients first into four or five subgroups for each of the three variables considering the corresponding optimal cutoff point and the sample size in each subgroup with the following values: 1) ≤4, 5–8, 9–13, 14–20, ≥21 for nLNE; 2) ≤4, 5–8, 9–12, 13–20 and ≥ 21 for nLNN; 3) 0, 0.01–0.05, 0.06–0.20 and ≥ 0.21 for LNR. The survival probability was calculated as a function of time for each of the subgroups. Then, the same patients were divided into two groups based on the subgroup value and the cutoff point indicated by the maximally selected rank statistics method to check the survival probability again. When the survival probability shows a clear difference between the two new groups, the optimal cutoff is found.

The statistical methods used for determining the cancer-specific survival (CSS) probability curve and the relationship between CSS and each of the main three variables are described here. The Kaplan-Meier method was used to estimate the esophageal CSS probability curve. Using the log-rank test, CSS probabilities between different subgroups were compared. The 5- and 10-year CSS probabilities, as well as the median follow-up time (the median observed survival time among all patients), were analyzed. Specifically, we used the multivariate Cox proportional cancer-specific hazard model to analyze the relationship between CSS and each of the three main variables after controlling for other secondary variables: tumor size, tumor grade, histology, year of diagnosis, and age at diagnosis. The effect size of risk factors was quantified by using a cancer-specific hazard ratio (HR) with 95% confidence intervals (95% CIs).

In addition, we performed sensitivity analysis to test the stability of the results by comparing hazard ratios including and excluding patients with tumor size > 20 cm and lymph nodes examined > 25 (126 patients in total), because these values are extreme values for T1 patients, and we cannot determine the accuracy of these extreme values. All statistical calculations were conducted by using R software (version 3.4.0).

## Results

### The demographic and tumor characteristics of T1 patients

**Fig. 1:**
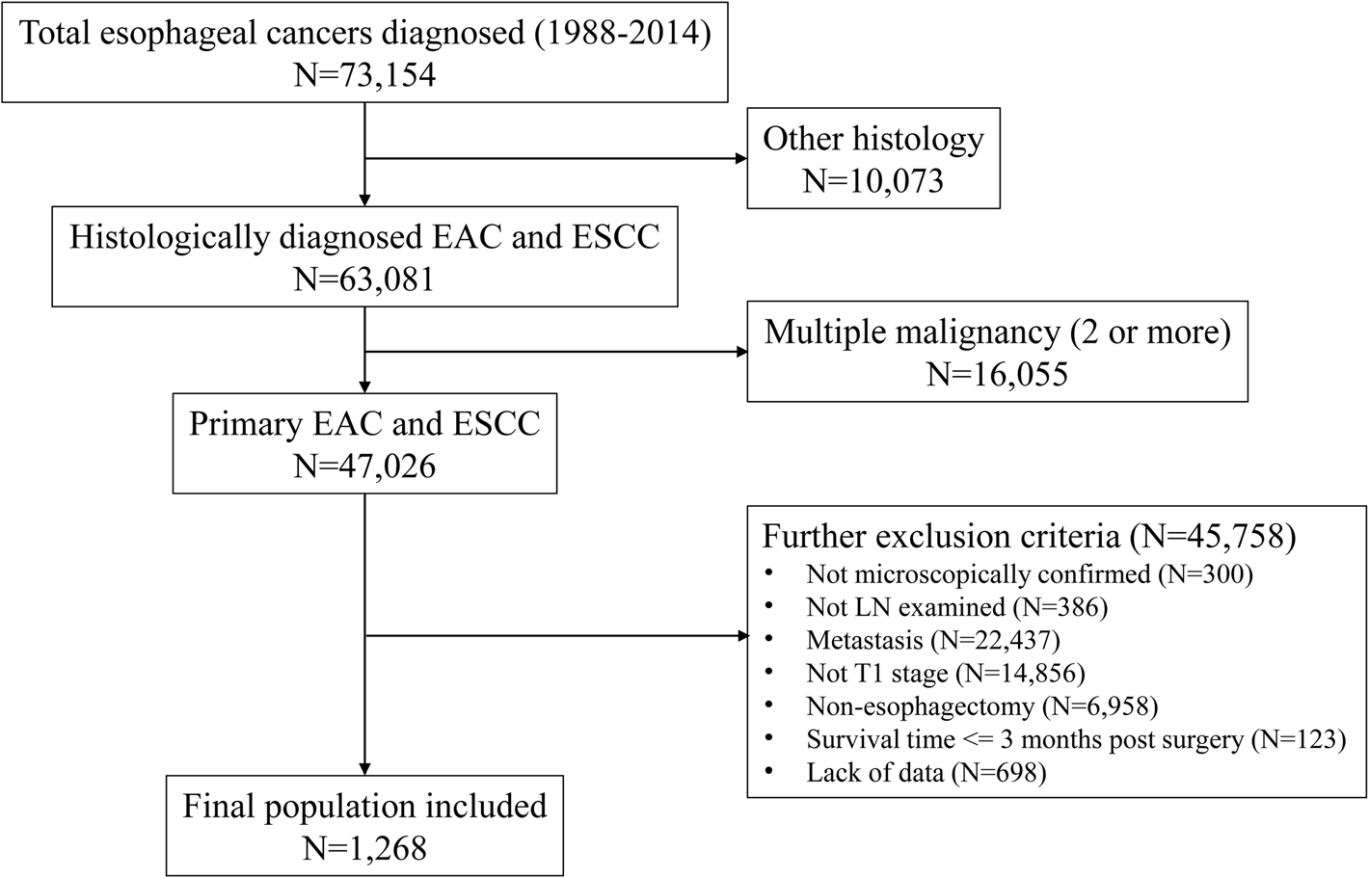
Road map of the patients included

We identified 1268 eligible patients with EAC or ESCC (Fig. 1). The demographic and tumor characteristics are presented in Table 1. Most patients were men (84.2%), and the median age of all patients was 63 years. The histologic type is mainly adenocarcinoma (79.9%). Approximately 18.0% are lymph node metastases (LN+), among which the median number of LN+ is 1. The prevalence of LN+ in EAC and ESCC is 17.7 and 18.4%, respectively. The median values of lymph nodes examined, negative lymph nodes and the LNR were 10, 10 and 0, respectively.

**Table 1:** Characteristics of esophageal cancer patients undergoing resection

Variable	All patients
**Age, years**	
Median, range	63 (20, 92)
< =68 (*n*, %)	901 (71.1%)
> 68 (*n*, %)	367 (28.9%)
**Sex**	
Male (*n*, %)	1068 (84.2%)
Female (*n*, %)	200 (15.8%)
**Tumor size, mm**	
Median, range	20 (0, 994)
< =25 (*n*, %)	763 (60.2%)
> 25 (*n*, %)	505 (39.8%)
**LNs status**	
LN+, *n* (%)	226 (17.8%)
LN-, *n* (%)	1042 (82.2%)
**Number of LNs examined**	
Median, range	10 (1, 90)
*Category 1*	
< =4 (*n*, %)	238 (18.8%)
5–8 (*n*, %)	303 (23.9%)
9–13 (*n*, %)	268 (21.1%)
14–20 (*n*, %)	239 (18.8%)
> =21 (*n*, %)	220 (17.4%)
*Category 2*	
< =13 (*n*, %)	809 (63.8%)
> 13 (*n*, %)	459 (36.2%)
**Number of LNs negative**	
Median, range	10 (0, 90)
*Category 1*	
< =4 (*n*, %)	262 (20.7%)
5–8 (*n*, %)	308 (24.3%)
9–12 (*n*, %)	221 (17.4%)
13–20 (*n*, %)	270 (21.3%)
> =21 (*n*, %)	207 (16.3%)
*Category 2*	
< =12 (*n*, %)	791 (62.4%)
> 12 (*n*, %)	477 (37.6%)
**LNR**	
Median, range	0 (0, 1)
*Category 1*	
0	1042 (82.2%)
0.01–0.05	26 (2.1%)
0.06–0.20	115 (9.1%)
> =0.21	85 (6.7%)
*Category 2*	
< =0.05 (*n*, %)	1068 (84.2%)
> 0.05 (*n*, %)	200 (15.8%)
**Grade**	
Well differentiated (*n*, %)	182 (14.4%)
Moderately differentiated (*n*, %)	613 (48.3%)
Poorly differentiated/Undifferentiated (*n*, %)	473 (37.3%)
**Histology**	
Adenocarcinoma (*n*, %)	1013 (79.9%)
Squamous cell carcinoma (*n*, %)	255 (20.1%)
**Year of diagnosis**	
1988–2014	1268

### Esophageal cancer-specific survival of T1 patients

The median follow-up time for these patients was 45 months (range, 4 to 258 months). There are 415 deaths resulting from esophageal cancer. The estimated 5- and 10-year CSS probabilities for all patients were 66.8 and 59.4%, respectively.

### Lymph node metastasis and esophageal cancer-specific survival

**Fig. 2:**
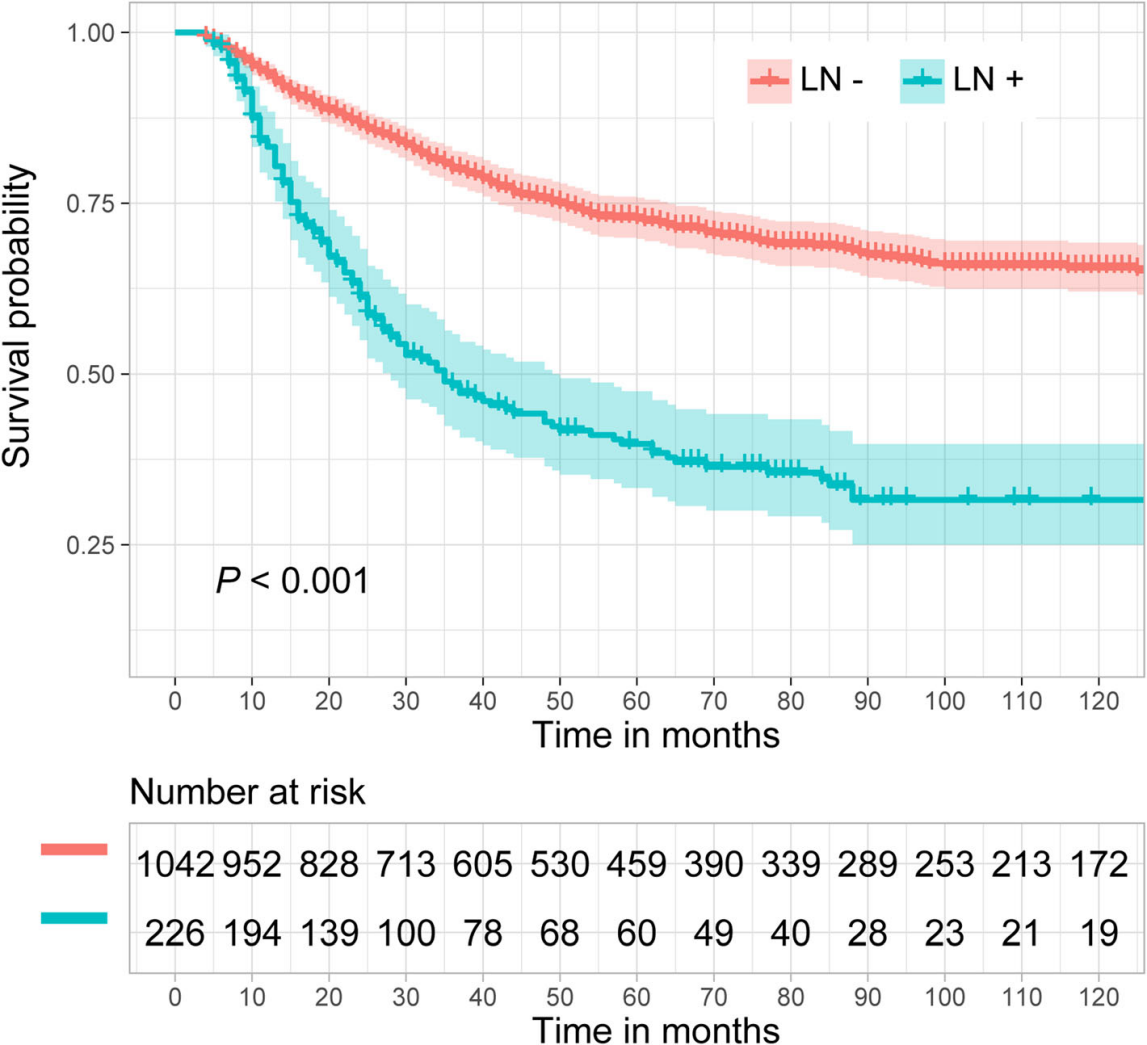
Esophageal cancer-specific survival curve with and without lymph node metastasis (LN+ vs LN-)

Figure 2 shows the CSS probability of esophageal cancer with and without lymph node metastasis (LN+ vs LN-). The estimated 5- and 10-year CSS probabilities for patients with lymph node metastasis were 39.8 and 31.5%, respectively, while they were 72.7 and 65.5% for patients without lymph node metastasis. This difference was significant (*P* < 0.001).

### nLNE and esophageal cancer-specific survival

**Fig. 3:**
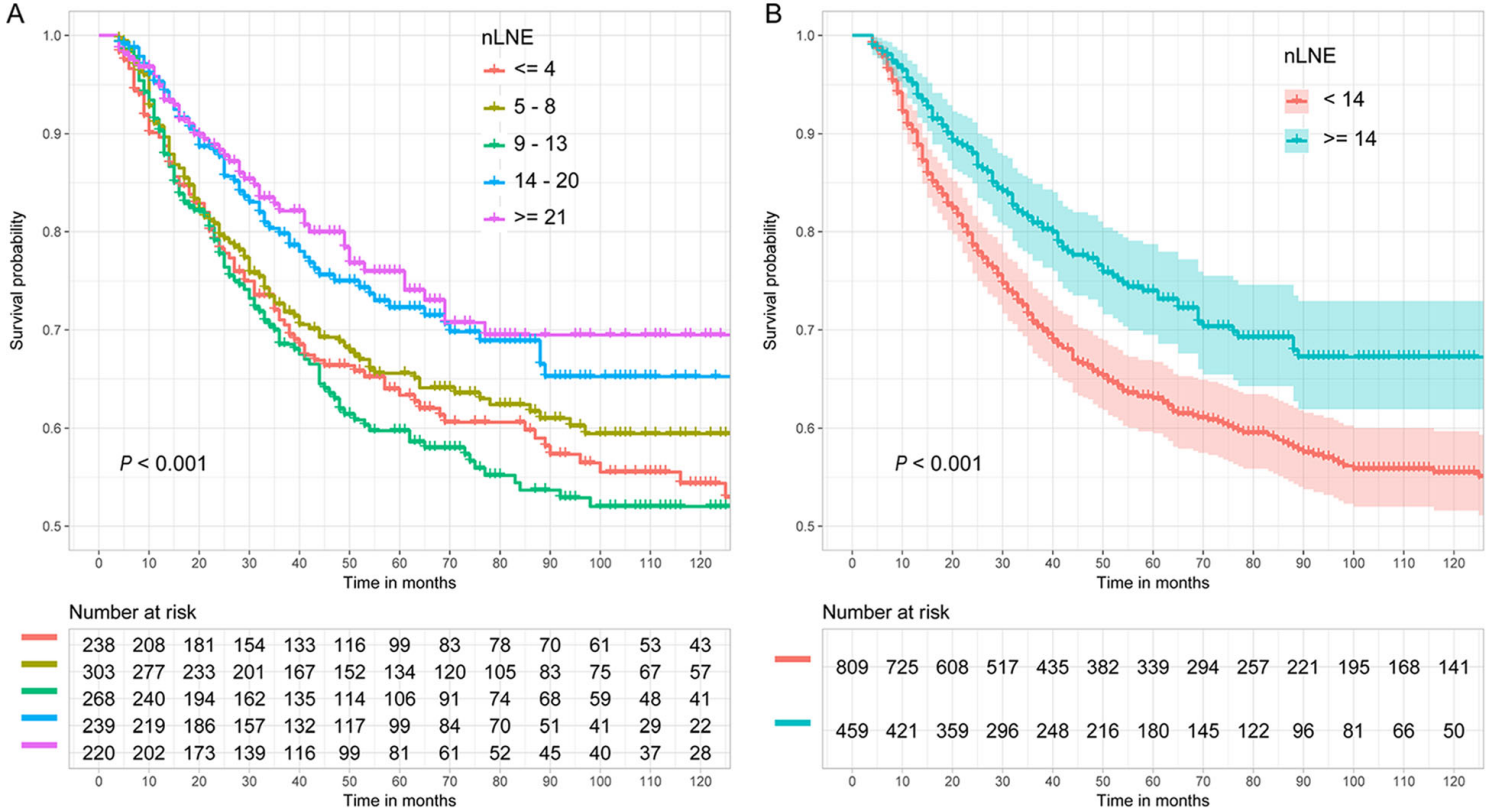
Esophageal CSS curve for different nLNEs based on two grouping methods. **a**: ≤4, 5–8, 9–13, 14–20 and ≥ 21; **b**: < 14 and ≥ 14

Figure 3 shows the CSS probability of esophageal cancer for different nLNEs. Figure 3a shows that the difference in CSS probability among the subgroups with different nLNEs (≤4, 5–8, 9–13, 14–20, and ≥ 21) was significant (*P* < 0.001). Generally, the CSS probability of patients with more lymph node resection was higher, except for the group with 9 to 13 lymph node resections. Figure 3b shows the CSS probability in the two groups separated based on a cutoff value of 13. The results indicate that the CSS probability of patients with ≥14 lymph nodes examined was significantly better than that of patients with < 14 lymph nodes examined (*P* < 0.001). The estimated 5- and 10-year CSS probabilities for patients with < 14 lymph nodes examined were 63.0 and 55.5%, respectively, compared with 74.0 and 67.2% for patients with ≥14 lymph nodes examined.

**Fig. 4:**
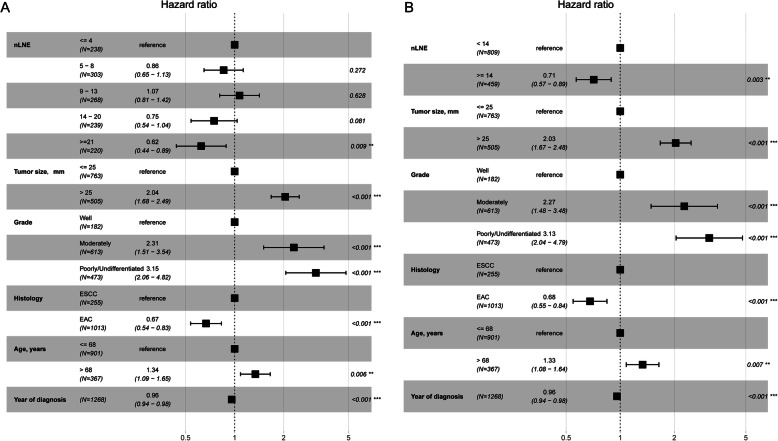
Cancer-specific hazard ratio for the nLNE **a**: ≤4, 5–8, 9–13, 14–20 and ≥ 21; **b**: < 14 and ≥ 14) on CSS probability after controlling for tumor size, tumor grade, histology, year of diagnosis, and age at diagnosis

Figure 4 shows the effect of nLNE on CSS probability after controlling for tumor size, tumor grade, histology, year of diagnosis, and age at diagnosis. Figure 4a corresponds to dividing the nLNE into five groups (≤4, 5–8, 9–13, 14–20, and ≥ 21), whereas Fig. 4b corresponds to dividing the nLNE into two groups (≥14 and < 14). Figure 4a shows that this main variable is statistically associated with CSS probability. The point estimation effects of nLNE among different groups are nonlinear, with the 9–13 group having the largest point estimation, although the difference between the 5–8 group, 9–13 group or 14–20 group and the ≤4 group is insignificant. Figure 4b shows that the cancer-specific death risk of patients with nLNE ≥14 is decreased by 29% compared to those with nLNE < 14 (HR 0.71, 95% CI: 0.57–0.89).

### nLNN and esophageal cancer-specific survival

**Fig. 5:**
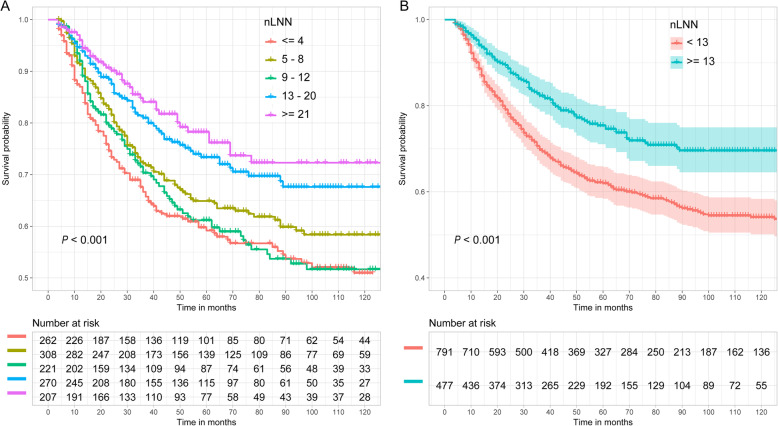
Esophageal CSS curve for different nLNNs based on the two grouping methods. **a**: ≤4, 5–8, 9–12, 13–20 and ≥ 21; **b**: < 13 and ≥ 13

Figure 5 shows the CSS probability of esophageal cancer for different nLNNs. Figure 5a shows that the difference in CSS probability among the subgroups with different nLNNs (≤4, 5–8, 9–12, 13–20, and ≥ 21) was significant (*P* < 0.001). Similar to Fig. 4a, generally the more negative lymph nodes there were, the higher the CSS probability for patients, except for the group with 9 to 12 negative lymph nodes. Figure 5b shows the CSS probability in two groups separated based on a cutoff value of 12. The results indicate that the CSS probability of patients with ≥13 negative lymph nodes is significantly better than that of patients with < 13 negative lymph nodes (*P* < 0.001). The estimated 5- and 10-year CSS probabilities for patients with < 13 negative lymph nodes were 62.0 and 54.1%, respectively, compared with 75.4 and 69.6% for patients with ≥13 negative lymph nodes.

**Fig. 6:**
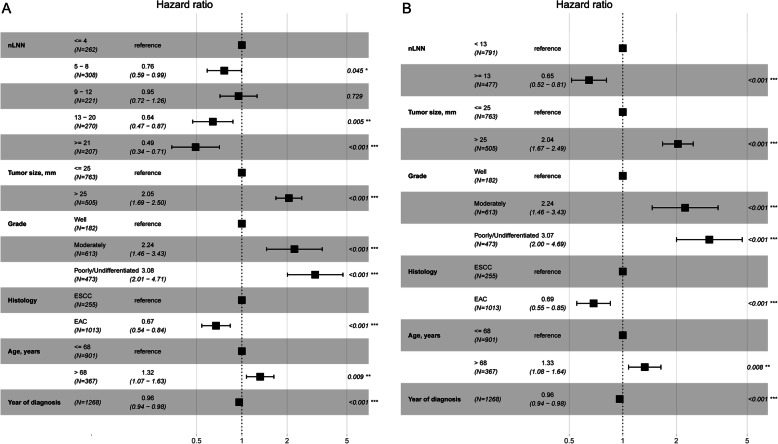
Cancer-specific HR for the nLNN **a**: ≤4, 5–8, 9–12, 13–20 and ≥ 21; **b**: < 13 and ≥ 13) on CSS probability after controlling for tumor size, tumor grade, histology, year of diagnosis, and age at diagnosis

Figure 6 shows the relationship between nLNN and CSS probability after controlling for other variables, including tumor size, tumor grade, histology, year of diagnosis, and age at diagnosis. Figure 6a corresponds to dividing the nLNN into four groups (≤4, 5–8, 9–12, 13–20, and ≥ 21), whereas Fig. 6b corresponds to dividing the nLNN into two groups (≥13 and < 13). Figure 6a shows that this main variable is statistically associated with CSS probability. The point estimation effects of nLNN among different groups are nonlinear, with the 9–12 group having the largest point estimation, although the difference between the 9–12 group and the ≤4 group is insignificant. Figure 6b shows that the cancer-specific death risk of patients with nLNN ≥13 was decreased by 35% compared to those with nLNN < 13 (HR 0.65, 95% CI: 0.52–0.81).

### LNR and esophageal cancer-specific survival

**Fig. 7:**
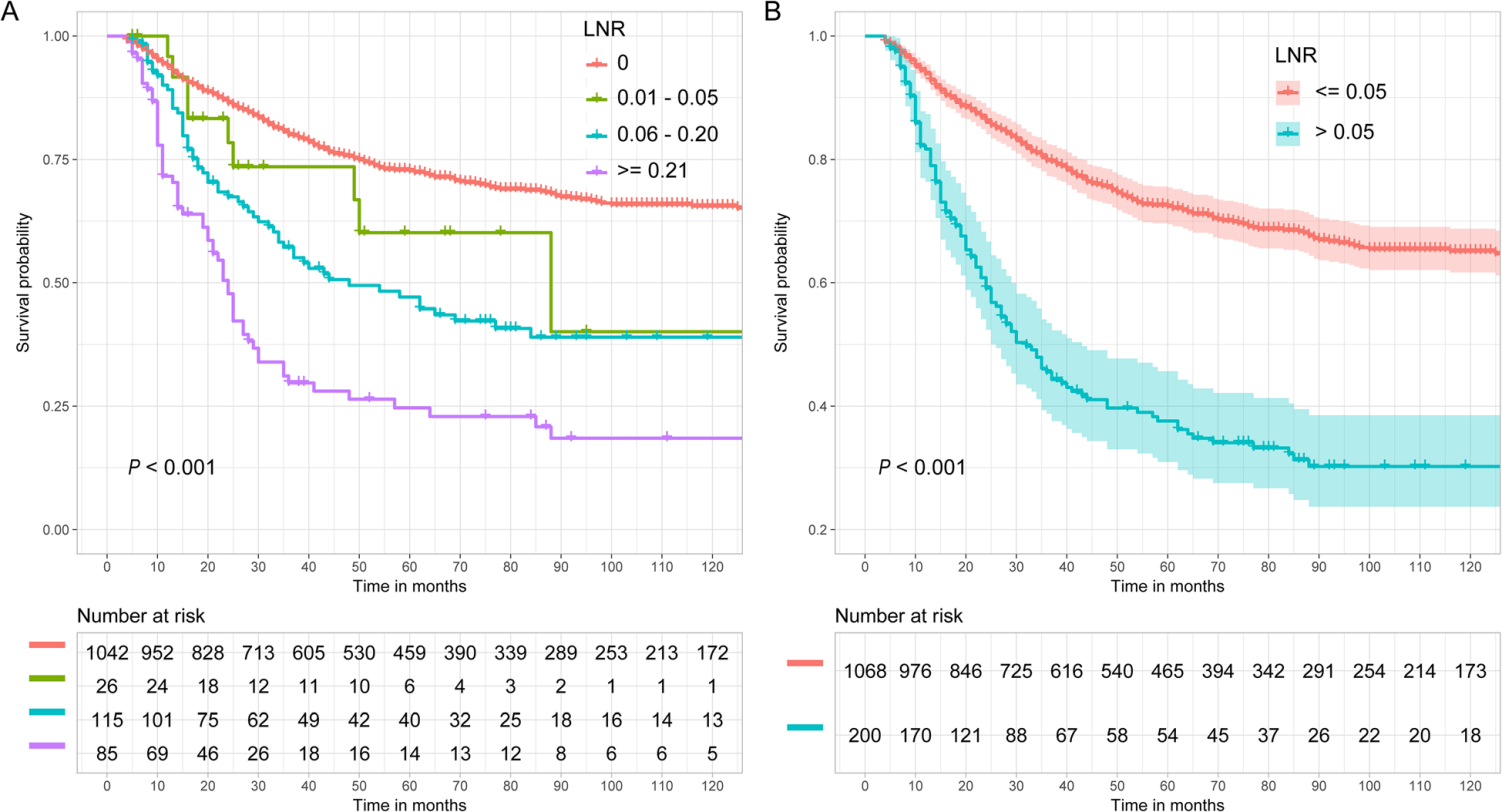
Esophageal CSS curve for different LNRs based on the two grouping methods. **a**: 0, 0.01–0.05, 0.06–0.20 and ≥ 0.21; **b**: ≤0.05 and > 0.05

Figure 7 shows the CSS probability of esophageal cancer for LNR. Figure 7a shows the CSS probability when dividing the patients into four groups (0, 0.01–0.05, 0.06–0.20, and ≥ 0.21). A significant difference is observed (*P* < 0.001). The larger the LNR is, the smaller the CSS probability. Figure 7b shows the CSS probability when dividing the patients into two groups: LNR ≤0.05 and > 0.05. The CSS probability in the ≤0.05 group was greatly improved compared with that in the > 0.05 group (*P* < 0.001). The estimated 5- and 10-year CSS probabilities for patients with LNR ≤0.05 were 72.4 and 65.1%, respectively, compared with 37.6 and 30.2% for patients with LNR > 0.05.

**Fig. 8:**
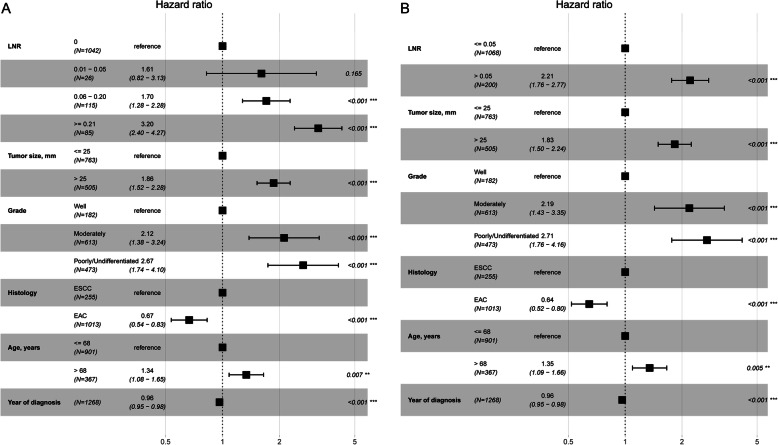
Cancer-specific HR for the LNR **a**: 0, 0.01–0.05, 0.06–0.20 and ≥ 0.21; **b**: ≤0.05 and > 0.05) on CSS probability after controlling for tumor size, tumor grade, histology, year of diagnosis, and age at diagnosis

Figure 8 shows the prognostic value of the LNR for CSS probability after controlling for tumor size, tumor grade, histology, year of diagnosis, and age at diagnosis. Figure 8a shows that the CSS probability in the 0 group was significantly different from that in the 0.06–0.20 and ≥ 0.21 groups but not significantly different from that in the 0.01–0.05 group. Figure 8b shows that the risk of cancer-specific death increased by 1.21 times (HR 2.21, 95% CI: 1.76–2.77) in the > 0.05 group compared to the ≤0.05 group.

### Sensitivity analysis

**Fig. 9:**
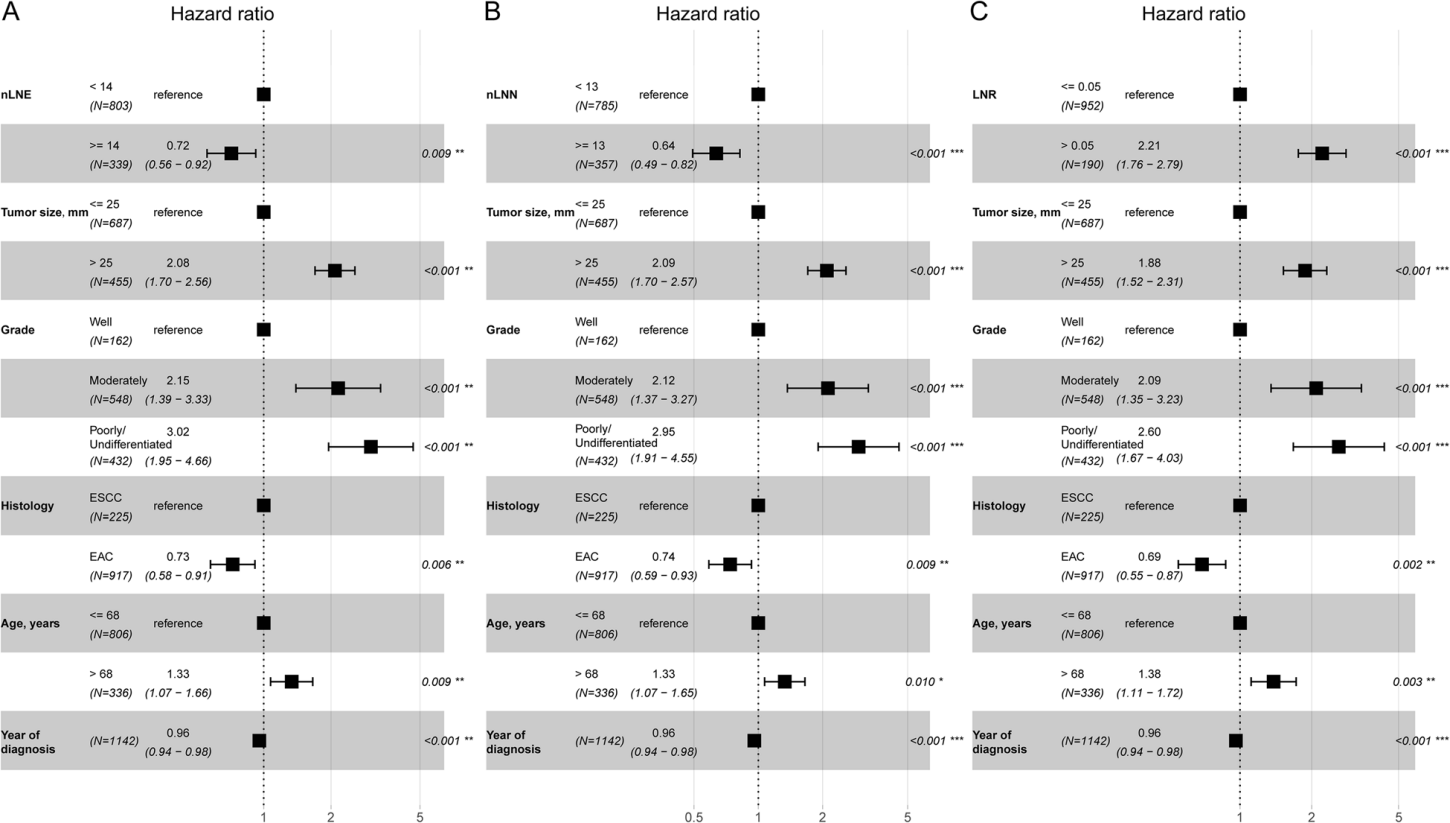
Sensitive analysis result of the cancer-specific hazard ratio for nLNE **a**, nLNN **b** and LNR **c** on CSS probability

The sensitivity analysis shows that the optimal cutoff points are not affected by excluding patients with extreme tumor size and nLNE values. The effect sizes of nLNE, nLNN and LNR on CSS are almost unchanged as shown in Fig. 9.

## Discussion

The study evaluated the effect of the extent of lymphadenectomy in T1 patients treated with esophagectomy. The esophageal cancer-specific survival (CSS) probability of patients in stage T1 is much improved with extensive lymphadenectomy. Our study evaluated three different variables, the number of lymph nodes examined, the number of negative lymph nodes and the LNR, to reveal the prognostic value of the extent of lymphadenectomy. We find that the thresholds of these three variables are 13, 12 and 0.05. The prognosis of patients was greatly improved in the groups with nLNE ≥14, nLNN ≥13, or LNR ≤0.05 compared to the other groups.

The 5-year survival probability in patients with advanced esophageal cancer can be less than 30%, but the prognosis of patients in the early stage is greatly improved [12, 13]. Lymph node metastasis is vital to the prognosis of patients in the early stage. Our study indicated that the CSS probability between patients with and without lymph node metastasis was markedly different in T1 patients. The CSS probability of patients without lymph node metastasis was approximately two times that of patients with lymph node metastasis.

However, lymphatic dissemination is chaotic and unpredictable, and occurs early in tumorigenesis. Because of the abundant lymph-capillary plexus in the lamina propria mucosa and submucosa of the esophagus, the potential for lymph node metastasis exists, even in patients with intramucosal cancer (T1a) [14, 15]. Therefore, a key step in the treatment of T1 patients is to identify metastatic lymph nodes during preoperative diagnosis. However, preoperative identification of patients with lymph node metastasis using the present modalities has many limitations. Thus, esophagectomy with lymphadenectomy is considered the first curative option for early-stage patients.

Most agree that lymphadenectomy does provide the benefit of accurate pathologic staging, but its effect on improving survival is debated. One study using data from the National Cancer Data Base (NCDB) found that examining an increased number of lymph nodes was associated with further survival improvements and recommended that 20 to 25 lymph nodes should be examined [8]. Another international multicenter study showed that the number of lymph nodes removed was an independent predictor of survival, and indicated a minimum of 23 lymph nodes to be removed to maximize the outcome of survival [9]. The study using the SEER database recommended that at least 30 lymph nodes should be removed to maximize survival [16]. One study indicated that the optimum lymphadenectomy was modulated by T stage. A study using Worldwide Esophageal Cancer Collaboration data recommended that the optimum lymphadenectomy was 10 lymph nodes for T1, 20 for T2, and ≥ 30 for T3/T4 [17]. However, some studies have indicated opposite conclusions on the prognostic value of the extent of lymph node excision. Lagergren et al. found that the extent of lymphadenectomy was not statistically associated with all-cause or cancer-specific mortality, and the results were the same after stratification by T category [7]. A Swedish nationwide population-based cohort study also provided evidence that more extensive lymphadenectomy did not improve survival after surgery and might even hamper survival in early T stage patients [10]; however, it should be noted that the median number of lymph nodes removed in the Swedish study was seven, which is relatively low and cannot effectively identify positive lymph nodes. Based on the controversial results, a recent meta-analysis confirmed that increased lymph node resection was associated with improved overall and cancer-free survival [18].

Similar to the relationship between nLNE and survival, some studies indicated that a higher number of negative lymph nodes was associated with better overall survival [19–21]. Schwarz et al. found that ≥15 negative lymph nodes was associated with the best overall survival [20]. Liu et al. suggested that at least 16 negative lymph nodes should be resected [22]. Yang et al. and Greenstein et al. recommended a minimum resection of 18 negative lymph nodes [21, 23]. We also found that the extent of lymphadenectomy was an independent factor and that improved survival was related to increasing nLNN. This agrees with some population-based studies that confirmed the relationship between the extent of lymphadenectomy and esophageal cancer survival. We further suggest a minimum lymph node dissection of 13 for nLNN for T1 stage patients.

**Fig. 10:**
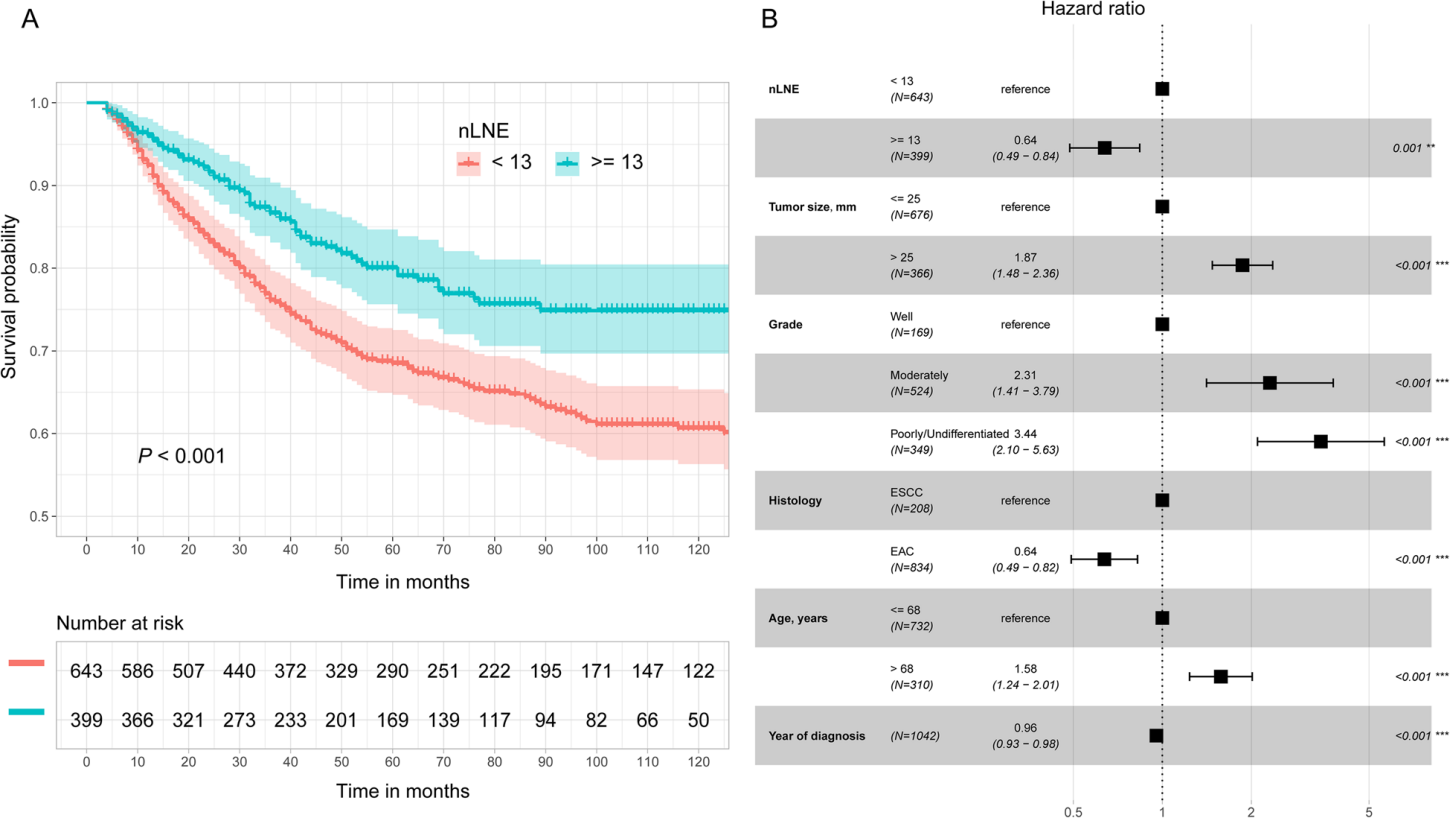
Esophageal CSS curve **a** and effect **b** of different lymph node resections in patients without lymph node metastasis

There are two possible explanations for our finding. One is improved survival due to stage migration [24]. As nLNE or nLNN increases, the probability of missing a positive lymph node and erroneously classifying as an earlier stage of cancer decreases. A second explanation is that the number of negative lymph nodes may contribute to a reduction in unrecognized tumor cells. Micrometastases have been shown to be present in up to 56% of N0 (with no positive lymph nodes examined) patients [25] and can even be up to 15% in early esophageal cancer [26]. In addition, studies have shown that micrometastases in esophageal cancer are associated with reduced survival and an increased risk of disease recurrence [26, 27]. For patients without lymph node metastasis, our study showed that the CSS probability with ≥13 lymph nodes dissected was much better than that with < 13 lymph nodes dissected (the estimated 5- and 10-year CSS probabilities for the ≥13 group vs < 13 group were 80.0% vs 68.5 and 74.9% vs 60.6%, *P* < 0.001 (Fig. 10a)). This corresponded to a 36% decrease in the risk of cancer-specific death in the group with ≥13 negative lymph nodes compared to the group with < 13 negative lymph nodes (Fig. 10b). This is consistent with some published studies indicating that high nLNN resection was independently associated with high disease-specific survival in patients with N0 esophageal cancer [22, 23]. The most likely explanation for the observed association between an increasing number of negative lymph node resections and improved survival is the clearance of micrometastasis foci. The current modalities have difficulty identifying small micrometastatic foci. Therefore, extensive lymphadenectomy may improve the prognosis by resecting residual cancer cells. In addition to absolute nLNE and nLNN, some studies also indicated that the LNR was an independent predictor of prognosis: the higher the LNR was, the worse the survival [7, 8, 28–31]. Other studies suggested that LNR < 0.1 or < 0.2 was associated with maximum survival benefit [8, 31].

Inconsistency exists regarding the cutoff values recommended by these earlier studies. One possibility is that the patients among different studies are heterogeneous. Some studies were based on EAC predominant populations [9, 16, 17, 23], and some were based on ESCC [19, 21, 22, 32–34]. Some included early-stage cancer [15, 23, 33], and others included T1-T4 but mainly advanced-stage cancer [8, 9, 16, 19–21, 32]. The other possibility is the heterogeneity of the method in grouping. Most studies grouped patients based on an arbitrarily defined cutoff points, such as quartiles, which is a subjective method [7, 19, 35, 36]. It is difficult to find the real effect of continuous variables. Relatively few studies have used more objective statistical methods to explore the relationship between the extent of lymph node excision and survival [8, 9, 17, 32].

In this study, we explored the relationship between the extent of lymph node resection (including nLNE, nLNN and LNR) and long-term CSS in T1 patients after esophagectomy and lymphadenectomy. It should be noted that surgeons cannot determine nLNN or LNR during the surgery; it can only be done by the pathological department after the surgery. Therefore, the nLNN and LNR indices have scientific value but cannot yet guide practice; however, nLNE can.

We used a large sample size to investigate the association between the extent of lymphadenectomy and cancer-specific death. This is advantageous for detecting any relatively moderate association. Cancer-specific death as the end point event can avoid unrelated causes of death, such as age-related death. There were many non-cancer-related deaths in T1 patients in our study (cancer-specific deaths vs all-cause deaths: 415 vs 595). Choosing cancer-specific death as the end point can reveal the true effect of the extent of lymph node resection on survival. The populations in our study were all T1 patients, which is homogenous. Therefore, the conclusion is specific to T1 patients.

This study has its limitations. First, the current AJCC TNM classification separates T1 patients into T1a and T1b based on the distinguished prognosis, but the information on tumor infiltration depth in the SEER database classify some patients into T1, but not specific into T1a and T1b. Second, there is no detailed and clear information regarding resection margin, comorbidity, surgical volume, surgical approach (open versus minimally invasive), surgical technique (Ivor-Lewis versus transhiatal esophagectomy or 2-field versus 3-field lymphadenectomy) and neoadjuvant chemotherapy and radiotherapy. However, neoadjuvant chemotherapy and radiotherapy are not the standard therapies for early-stage cancer, especially for the patients in our study with N0 disease, which accounted for 82.2%. Third, surgeons usually perform lymph nodes dissection according to the histology (EAC and ESCC) and tumor location (cervical, thoracic and abdominal). In our analysis, the ideal case would be to conduct subgroup analyses considering histology and tumor location. However, this would require a larger sample size than what we have in order to achieve the statistical power for a meaningful result. This may be a future study when large enough sample size becomes available. Therefore, readers should view the result of this study as a scientific progress report, not a golden standard for guiding the surgeons, such as 14 as the recommended minimum number of lymph nodes that need to be removed. Furthermore, our study is a retrospective observational study. The results should be verified by conducting a prospective study.

## Conclusions

In summary, the prognosis of early esophageal cancer has largely improved compared to that of advanced cancer. Greater extensive lymphadenectomy can improve cancer-specific survival in T1 patients. This study finds statistically that the minimum number of lymph nodes that need to be removed is 14. The number of negative lymph nodes and the lymph node ratio also have prognostic value after lymphadenectomy among T1 stage patients. This study adds to the evidence knowledge pool for improving cancer-specific survival in patients with T1 esophageal cancer. As future available sample size increases, the accuracy from statistical modelling will also be improved. Therefore, the exact number of lymph node dissection in practices should still be determined based on the guidance from each country, such as tumor location and histology.

## Data Availability

The datasets generated and/or analysed during the current study are available in the Surveillance, Epidemiology and End Results (SEER) Program databases (https://seer.cancer.gov/data/).

## Abbreviations

AJCC: The American Joint Committee on Cancer
TNM: Tumor Node Metastasis
NCCN: National Comprehensive Cancer Network
nLNE: Number of lymph nodes examined
nLNN: Number of negative lymph nodes
LNR: Lymph node ratio
CSS: Cancer-specific survival
SEER: Surveillance, Epidemiology and End Results
EAC: Esophageal adenocarcinoma
ESCC: Esophageal squamous cell carcinoma
HR: Hazard ratio
CIs: Confidence intervals
NCDB: National Cancer Data Base
